# Distribution diversity and expression regulation of class 1 integron promoters in clinical isolates of *Morganella morganii*

**DOI:** 10.3389/fmicb.2024.1459162

**Published:** 2024-10-18

**Authors:** Ye Yang, Hui Zhang, Rongqing Zhao, Xuedan Qiu, Jinglu Ye, Wenjun Lu, Qingcao Li, Guangliang Wu

**Affiliations:** ^1^Department of Clinical Laboratory, The Affiliated LiHuiLi Hospital of Ningbo University, Ningbo, China; ^2^Department of Clinical Laboratory, Ninghai County Chengguan Hospital, Ningbo, China; ^3^Department of Intensive Care Units, The Affiliated LiHuiLi Hospital of Ningbo University, Ningbo, China; ^4^Department of Clinical Pharmacy, The Affiliated LiHuiLi Hospital, Ningbo University, Ningbo, China

**Keywords:** *Morganella morganii*, integron, promoter, expression regulation, homology

## Abstract

**Background:**

*Morganella morganii* is an emerging nosocomial opportunistic pathogen with increasing multidrug resistance. Antibiotic resistance, driven primarily by the horizontal transfer of resistance genes, has become a global health crisis. Integrons, mobile genetic elements, are now understood to facilitate the transfer of these genes, contributing to the rapid proliferation of resistant strains. Understanding the regulatory role of integrons in drug resistance gene expression is crucial for developing novel strategies to combat this pressing public health issue.

**Objective:**

To investigate the distribution of promoter types in the variable regions of class 1 integrons isolated from clinical isolates of *M. morganii* and their regulatory role in the expression of downstream drug resistance gene cassettes.

**Methods:**

Ninety seven clinical isolates of *M. morganii* were screened for the presence of class 1 integrons (*intI1*) using polymerase chain reaction (PCR). Gene cassettes within the variable regions of positive isolates were characterized, and the gene cassette promoter Pc variants and downstream auxiliary promoter P2 were identified. Enterobacterial repetitive intergenic consensus (ERIC)-PCR was employed for homology analysis. Recombinant plasmids containing different variable region promoters and gene cassettes were constructed to evaluate drug resistance genes and integrase (*intI1*) expression levels using reverse transcription-quantitative PCR (RT-qPCR) and antimicrobial susceptibility testing.

**Results:**

Of the clinical isolates, 28.9% (*n* = 28/97) were positive for class 1 integrons. 24.7% (*n* = 24/97) of these isolates carried gene cassettes encoding resistance to aminoglycosides and trimethoprim. Three Pc promoter types (PcH1, PcS, and PcW) were identified, while all P2 promoters were inactive with a 14-base pair spacing between the −35 and −10 regions. ERIC-PCR analysis classified the integron-positive strains into 6 genotypes, with high consistency in promoter types and gene cassettes within each genotype. RT-qPCR and antimicrobial susceptibility testing demonstrated that strong promoters significantly enhanced the expression of downstream drug resistance gene cassettes compared to weak promoters. Additionally, RT-qPCR revealed a negative correlation between *intI1* expression and Pc promoter strength.

**Conclusion:**

Class 1 integrons are prevalent in *M. morganii*. The promoter types within these integrons are diverse, and promoter strength is closely linked to downstream gene cassette expression. Integron-positive strains exhibit high homology, suggesting horizontal gene transfer and dissemination in clinical settings.

## 1 Introduction

*Morganella morganii*, a member of the *Enterobacteriaceae* family, has historically been detected infrequently in clinical settings (Agrawal et al., 2021). While traditionally associated with urinary tract infections, wound infections, and bloodstream infections (Laupland et al., 2022; Shi et al., 2022; Gameiro et al., 2023; Kvopka et al., 2023; Li et al., 2023; Yeşil et al., 2023; Alsaadi et al., 2024), its clinical isolation rate has been rapidly increasing in recent years, leading to severe invasive infections (Zaric et al., 2021; Alelyani et al., 2022; Behera et al., 2023a; Elmi et al., 2024). Due to its high mortality rates in specific patient populations, the World Health Organization has designated *M. morganii* as a globally prioritized pathogen (Behera et al., 2023b). Some clinically isolated strains of *M. morganii* have acquired resistance to multiple antibiotics through the carriage of various drug resistance genes, posing serious challenges for clinical infection control (Liu et al., 2016). Horizontal transfer of drug resistance genes, facilitated by mobile genetic elements such as integrons, is a common mechanism for acquiring resistance in bacteria (Leverstein-van Hall et al., 2002; von Wintersdorff et al., 2016; Coluzzi et al., 2023). However, the role of integrons in *M. morganii* resistance has been largely understudied.

Current evidence suggests that integrons, typically consisting of three main components: a 5′ conserved region, a variable region containing various drug resistance gene cassettes, and a 3′ conserved region that varies with integron type, play a pivotal role in the horizontal transfer of drug resistance genes among bacteria (Hall and Stokes, 1993). They are natural cloning and expression vectors, capturing and disseminating gene cassettes through site-specific recombination (Hall and Collis, 2006). One of the most extensively studied integron types is class 1 integrons, which include the Pc promoter in the 5′ conserved region. As most gene cassettes lack promoters, the Pc promoter plays a crucial role in regulating the expression of downstream gene cassettes within integrons (Hanau-Berçot et al., 2002; Tseng et al., 2014). In some cases, the Pc promoter complements the auxiliary promoter P2, forming a Pc-P2 dual promoter configuration that further influences gene regulation (Stokes et al., 1997). The strength of the promoter, determined by transcriptional dominance, varies among different Pc variants defined by their −35 and −10 hexamer sequences. The most common Pc promoters in clinical and natural settings are Pc strong (PcS), Pc weak (PcW), Pc Hybrid 1 (PcH1), and Pc Hybrid 2 (PcH2) (Fonseca and Vicente, 2022). The transcription level of genes within integrons largely depends on the regulatory role of these promoters (Novačić et al., 2022), influenced by factors such as promoter strength and proximity to the gene cassette (Jacquier et al., 2009). Integron-positive *M. morganii* can express resistance to relevant drug resistance genes within the integron, and the expression of these genes is primarily dependent on the regulation of the variable region Pc promoter. The horizontal transmission of drug resistance genes between different bacterial strains, facilitated by integron-carrying *M. morganii*, poses a significant challenge in clinical settings. To investigate the relationship between variable region promoters of class 1 integrons in *M. morganii* and the regulation of drug resistance gene expression, this study analyzed antibiotic resistance data from non-duplicated clinical isolates collected from November 2015 to August 2021 at the Affiliated LiHuiLi Hospital of Ningbo University. The findings of this study provide valuable insights into the mechanism and expression regulation of drug resistance genes in integron-positive *M. morganii*, which has significant clinical implications for the prevention and treatment of this rare pathogen.

## 2 Materials and methods

### 2.1 Strains and plasmids

Ninety seven non-duplicated clinical isolates of *M. morganii* were collected from urine, bile, and other specimen types at the Affiliated LiHuiLi Hospital of Ningbo University between November 2015 and August 2021. Strains lacking complete clinical data were excluded. This study was approved by the Medical Ethics Committee of the Affiliated LiHuiLi Hospital of Ningbo University. *Escherichia coli* DH5α served as the integron-negative control strain, *Proteus mirabilis* 47437 as the class 1 integron-positive control strain, *E. coli* JM109 as the competent strain, and *E. coli* ATCC25922 as the control strain for antimicrobial susceptibility testing. Plasmid pACYC184 was used as the cloning and expression vector. All strains and plasmids were maintained in our laboratory.

### 2.2 Detection of integron-positive strains and variable gene cassettes

DNA templates were extracted from experimental strains using the boiling method. PCR amplification was performed using *intI1* screening primers (intF & P2R, Table 1) at an annealing temperature of 55°C. PCR products were analyzed by agarose gel electrophoresis to identify positive bands. Sequencing analysis was conducted to confirm the presence of class 1 integrons in the positive strains. Gene cassettes within the variable regions of class 1 integron-positive strains were amplified using specific primers (5CS & 3CS, Table 1) at an annealing temperature of 55°C. Positive bands were visualized by agarose gel electrophoresis, and sequencing of the PCR products was followed by comparison with the BLAST database to determine the composition of variable gene cassettes in these strains.

**Table 1:** Oligonucleotide primers used in this study.

Primer	Sequence (5′ → 3′)	Target gene	References
intF	CCAAGCTCTCGGGTAACATC	*intI1*	Lu et al. (2022)
P2R	GCCCAGCTTCTGTATGGAAC		Lu et al. (2022)
5CS	GGCATCCAAGCAGCAAG	Variable region	Lu et al. (2022)
3CS	AAGCAGACTTGACCTGA		Lu et al. (2022)
ERIC2	AAGTAAGTGACTGGGGTGAGCG	ERIC-PCR	Lu et al. (2022)
dfrA17R	GGATATCAGGACCACTACCGATTAC	Pc, P2	This study
aac(6′)R	GAAGCCAACCCATCAGACAG		This study
dfrA12R	GATAAATGCGTACTGATTCCGAGTTC		This study
aadBR	CAATGTGACCTGCGTTGTGTC		This study
dfrA32R	GGAATATCAGGACCACTACCGATTAC		This study
aadA1R	CTACCTCTGATAGTTGAGTCGATACTTC		This study
aacA4R	CAACGTGTTTTGAAGGCCTTC		This study
dfrA7R	GAGTAACTGCTCACCTTTTGCTG		This study
dfrA15R	CTGCCATTAGTGATAGTTTCACGATAC		This study
aadA2R	GCTTAGCACCTCTGATAGTTGGTTC		This study
aadF2	CGATGAGCGAAATGTAGTG	*aadA2*	Wei (2010)
aadR2	AAGACGGGCTGATACTGG		Wei (2010)
QCMF	GGAGTGAATACCACGACG	*cat*	Wei (2010)
QCMR	GGATTGGCTGAGACGAA		Wei (2010)

### 2.3 The distribution of promoters and their relationship with resistance in class 1 integron-positive strains

To identify Pc and P2 promoters in class 1 integron-positive strains, reverse primers were designed based on the sequences of the first gene cassettes at the 5′-conserved sequence (5CS) end, in conjunction with the forward primer intF (Table 1). PCR was conducted with annealing temperatures ranging from 52°C to 56°C for 35 cycles. Amplified products were visualized by agarose gel electrophoresis, and positive bands were sequenced to determine the promoter types. Clinical isolates were categorized according to their integron promoter types. A retrospective analysis of antibiotic resistance data was performed for each group of isolates, followed by a comparative analysis to evaluate differences in antibiotic resistance profiles among the various integron promoter-based groups.

### 2.4 ERIC-PCR detection of integron-positive strains

Enterobacterial repetitive intergenic consensus (ERIC) sequences are non-coding and highly conserved regions originally discovered in Enterobacteriaceae. Enterobacterial repetitive intergenic consensus-polymerase chain reaction (ERIC-PCR) involves the design of primers based on the conserved ERIC region for PCR amplification. The number and size of ERIC bands in the bacterial genome are then determined, and the degree of bacterial relatedness is calculated for typing purposes. Compared to other typing methods, ERIC-PCR is a popular choice due to its lower cost and faster operation (Meacham et al., 2003; Banoub et al., 2022). In the present study, integron-positive strains were subjected to ERIC-PCR analysis (using primer ERIC2, Table 1, annealing temperature 40°C, 40 cycles) to assess homology. Positive bands and their positions on agarose gel electrophoresis of PCR products were recorded, photographed, and compiled into a matrix table. NTsys 2.10e software was used to generate a clustering dendrogram. Additionally, integron-positive strains were genotyped, and homology analysis was conducted in conjunction with Pc promoter and drug resistance gene cassettes in the variable region.

### 2.5 Constructing strains containing recombinant plasmids

**Figure 1:**
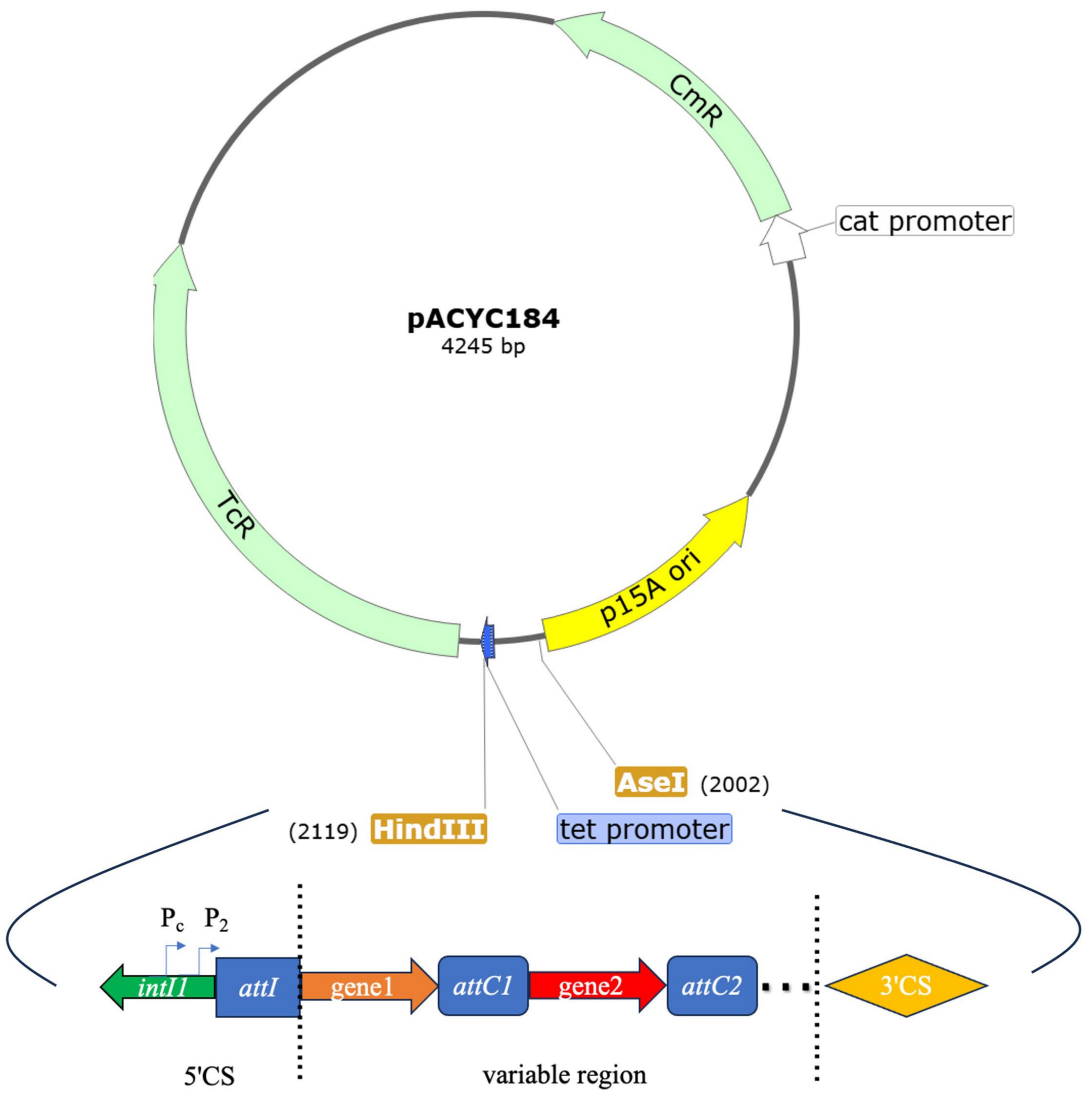
pACYC184 plasmid map and the sites of enzyme digestion HindIII and AseI. The pACYC184 plasmid map shows the positions of HindIII and AseI. The *tet* promoter has been disrupted by enzymatic cleavage. The map of the recombinant gene fragment that was integrated into the digested plasmid: the map includes the complete sequence from *intI1* to 3CS. *tet*: tetracycline resistance gene, *cat*: chloramphenicol resistance gene.

Two sets of recombinant plasmids were constructed based on different promoter types and gene cassettes within the variable region. The first set employed various variable region promoters while maintaining the same gene cassette, while the second set utilized the same variable region promoter and gene cassette but varied the distance from the Pc promoter. The pACYC184 plasmid, which confers chloramphenicol resistance, served as the vector. HindIII and AseI restriction enzymes were chosen to digest the plasmid, replacing the *tet* promoter to avoid interference with the integron promoters in the recombinant gene fragment (Figure 1). The larger fragment of the digested plasmid was then purified. A synthetic gene fragment, encompassing the integrase *intI1* to 3CS region (including the variable region promoter), was ligated to this purified plasmid fragment. The resulting recombinant plasmids were confirmed through sequencing and subsequently transformed into competent *E. coli* JM109 cells.

### 2.6 RT-qPCR and antimicrobial susceptibility testing of constructed strains

Total RNA was extracted from the logarithmic growth phase of the constructed strains. The extracted RNA was purified and subjected to reverse transcription (RT) to obtain cDNA templates, which were then diluted to appropriate concentrations for subsequent quantitative PCR (qPCR) detection. qPCR was performed using specific primers targeting the genes of interest in the constructed strains. The pACYC184 plasmid-encoded *cat* gene was used as an internal reference gene. Relative expression levels of genes were calculated using the 2^−ΔΔCt^ method. The expression levels of the target gene and the integrase gene *intI1* were analyzed to compare transcriptional differences among the various constructed strains.

Antimicrobial susceptibility testing and interpretation of experimental results was conducted following Clinical and Laboratory Standards Institute (CLSI) guidelines (M-100, Ed 2022) (CLSI, 2022). The broth microdilution method was used to determine the minimum inhibitory concentrations (MICs) of antibiotics mediated by drug resistance gene cassettes in the variable region. Chloramphenicol (mediated by the internal reference gene *cat*) and tetracycline (mediated by gene *tet*) were also tested in each constructed strain. *E. coli* ATCC25922 served as the quality control strain for susceptibility testing, and *E. coli* JM109 was used as the negative control strain. The Kirby-Bauer (K-B) disk diffusion method was employed to validate the results obtained from MIC determination, allowing for a comparison of resistance differences among the constructed strains.

### 2.7 Statistical analysis

In this study, the comparison of drug resistance rates between different groups was analyzed using Fisher’s exact test (two-sided) with SPSS 25.0 software, considering a significance level of *p* < 0.05. Gene relative expression levels in qPCR were analyzed using one-way ANOVA for multiple comparisons among strains with GraphPad Prism 8.0 software, using a significance level of *p* < 0.05.

## 3 Results

### 3.1 Detection of class 1 integrons and gene cassettes of variable regions

We identified 28 out of 97 clinical isolates of *M. morganii* as positive for *intI1*, resulting in a positivity rate of 28.9% (Supplementary material: Sequence of *intI1*). Among these *intI1*-positive isolates, 10 different types of gene cassettes were amplified, while 4 isolates did not amplify any gene cassettes (Supplementary material: Sequence of variable region). The amplified gene cassettes primarily conferred resistance to aminoglycosides and trimethoprim, as shown in Table 2.

**Table 2:** Variable region gene cassette distribution.

Number of strains	Length (bp)	Gene cassette of variable region
9	1,393	*dfrA17-aadA5*
3	474	*dfrA17*
3	792	*aadA2*
2	1,697	*dfrA12-aadA2*
2	474	*dfrA7*
1	1,442	*aacA4-orfD-aadB*
1	474	*dfrA15*
1	2,966	*aadB-aadA2-cmlA6*
1	2,763	*dfrA32-ereA1-aadA2*
1	1,149	*aac(6′)-Ib-cr-arr-3*
4	–	ND

### 3.2 Distribution of promoters and their relationship with antibiotic resistance in *Morganella morganii*

**Figure 2:**
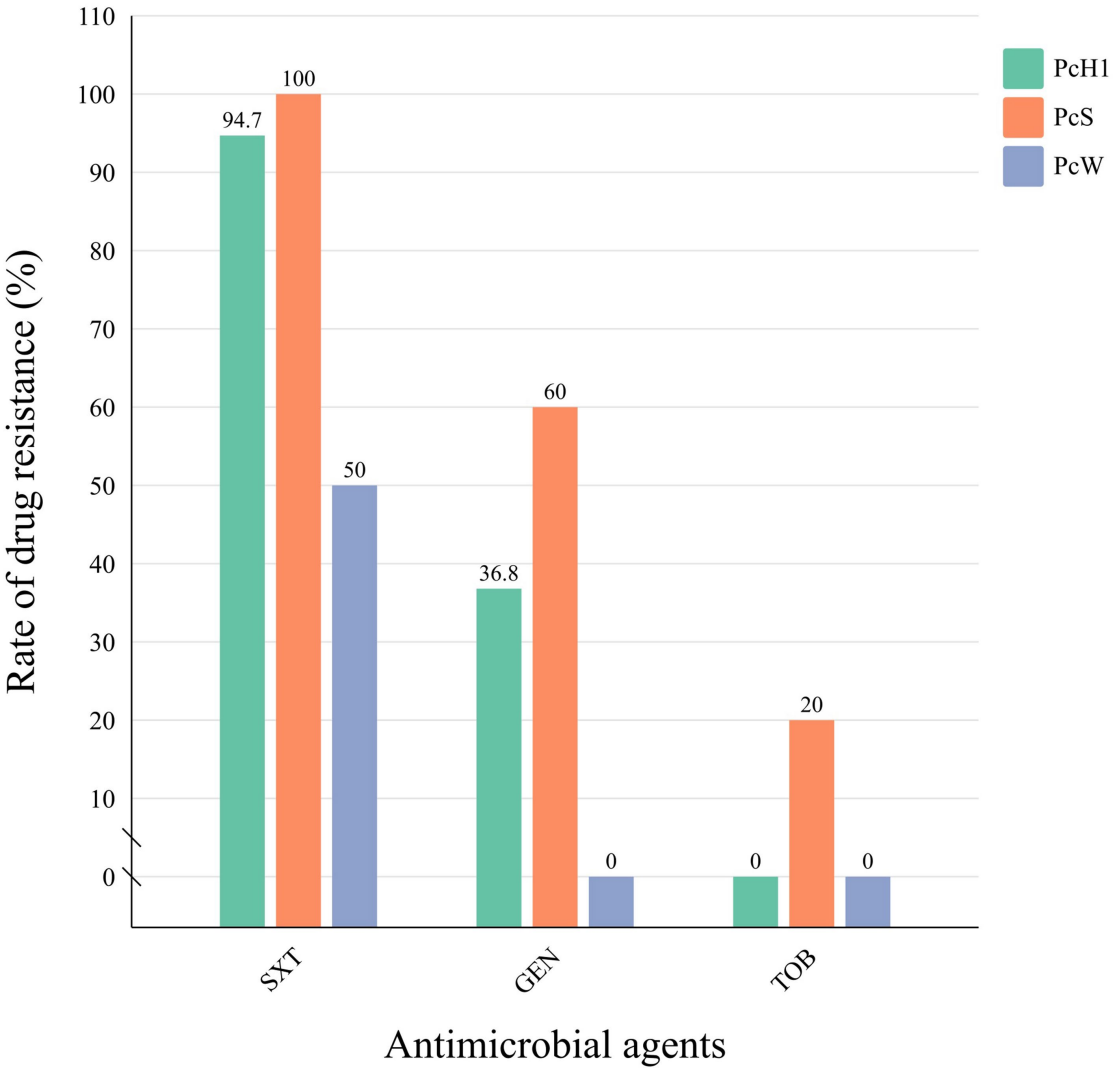
The comparison of antibiotic resistance rates among different promoter groups in *Morganella morganii* isolates. STX, cotrimoxazole; GEN, gentamicin; TOB: tobramycin.

Among the 28 strains positive for class 1 integrons, three types of Pc promoters were detected: PcH1 (*n* = 19), PcS (*n* = 5), and PcW (*n* = 4). PcS exhibited stronger promoter activity, PcW showed weaker activity, and PcH1 demonstrated intermediate strength. All downstream P2 promoters were inactive types, spaced 14 base pairs apart from the −35 to −10 regions (Supplementary material: Sequence of promoter). Based on the different types of variable region promoters in the integrons, the positive strains were categorized into three groups. Retrospective analysis of drug resistance data was conducted among strains containing different types of variable region promoters. Although there were no significant differences in resistance rates to cotrimoxazole, gentamicin, or tobramycin among the groups (*p* > 0.05), strains in the PcS group exhibited higher resistance rates to all antibiotics compared to the PcH1 and PcW groups, while the PcW group demonstrated the lowest resistance rates among the three groups (Figure 2).

### 3.3 ERIC-PCR typing results

**Figure 3:**
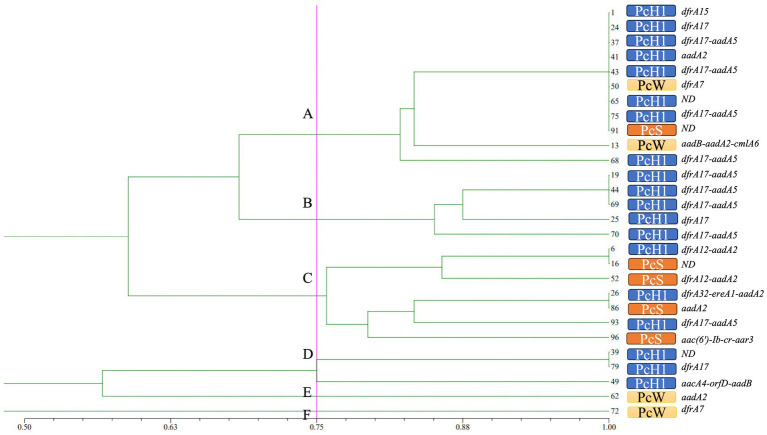
The dendrogram of ERIC-PCR. The solid magenta line represents the 75% homologous gene typing reference line. On the right side of the dendrogram are serial numbers of strains, variable region promoter Pc and gene cassettes of integrons successively.

ERIC-PCR fingerprinting of the 28 class 1 integron-positive strains was encoded and organized into a matrix table (Supplementary Figure S1; ERIC-PCR electrophoretogram). NTsys 2.10e software was used to generate a dendrogram with a reference line set at 75% similarity (Bakhshi et al., 2018). Based on this analysis, the positive strains were classified into 6 distinct genotypes (A, B, C, D, E, F). Genotype A was the most prevalent, with 11 strains, followed by genotype C with 7 strains. The predominant type of variable region promoter Pc was PcH1. Gene cassette dfrA was the dominant gene in the variable region of type A isolates, which exhibited high resistance to trimethoprim. Type B isolates primarily carried the PcH1/dfrA17-aadA5 combination, associated with resistance to aminoglycosides and trimethoprim. Type C isolates predominantly had the PcS promoter and showed relatively high resistance rates to various antibiotics. The distribution of gene cassettes was concentrated, primarily consisting of aminoglycoside and trimethoprim drug resistance gene cassettes. Specific details are illustrated in Figure 3.

### 3.4 Construction of strains containing recombinant plasmids

Based on five class 1 integron-positive strains of *M. morganii* (MM-6, MM-26, MM-41, MM-62, MM-86), each containing the *aadA2* gene cassette in the variable region, the Pc promoters were PcH1, PcH1, PcH1, PcW, and PcS, respectively. The P2 sequences were uniformly inactive, spanning 14 base pairs between the −35 and −10 regions. Gene fragments containing integrase, promoters, variable region gene cassettes, and inter-cassette sequences were synthesized according to actual sequencing results (Supplementary material: The gene synthesis of target segment in recombinant plasmids). These gene fragments were used to recombine plasmids, resulting in the creation of five recombinant strains named JM-6, JM-26, JM-41, JM-62, and JM-86, as listed in Table 3.

**Table 3:** Distribution of variable region promoters and gene cassettes of 5 isolates and 5 constructed strains.

Strains	Pc		Pc	The activity of P2	Variable region gene
	−35 region	−10 region			
MM-6	TGGACA	TAAACT	PcH1	Inactive	*dfrA12-aadA2*
MM-26	TGGACA	TAAACT	PcH1	Inactive	*dfrA32-ereA1-aadA2*
MM-41	TGGACA	TAAACT	PcH1	Inactive	*aadA2*
MM-62	TGGACA	TAAGCT	PcW	Inactive	*aadA2*
MM-86	TTGACA	TAAACT	PcS	Inactive	*aadA2*
JM-6	TGGACA	TAAACT	PcH1	Inactive	*dfrA12-aadA2*
JM-26	TGGACA	TAAACT	PcH1	Inactive	*dfrA32-ereA1-aadA2*
JM-41	TGGACA	TAAACT	PcH1	Inactive	*aadA2*
JM-62	TGGACA	TAAGCT	PcW	Inactive	*aadA2*
JM-86	TTGACA	TAAACT	PcS	Inactive	*aadA2*

*aadA2: gene of adenosine transferase for aminoglycosides.*

### 3.5 Real-time quantitative PCR results of *aadA2* and *intI1* in constructed strains

**Figure 4:**
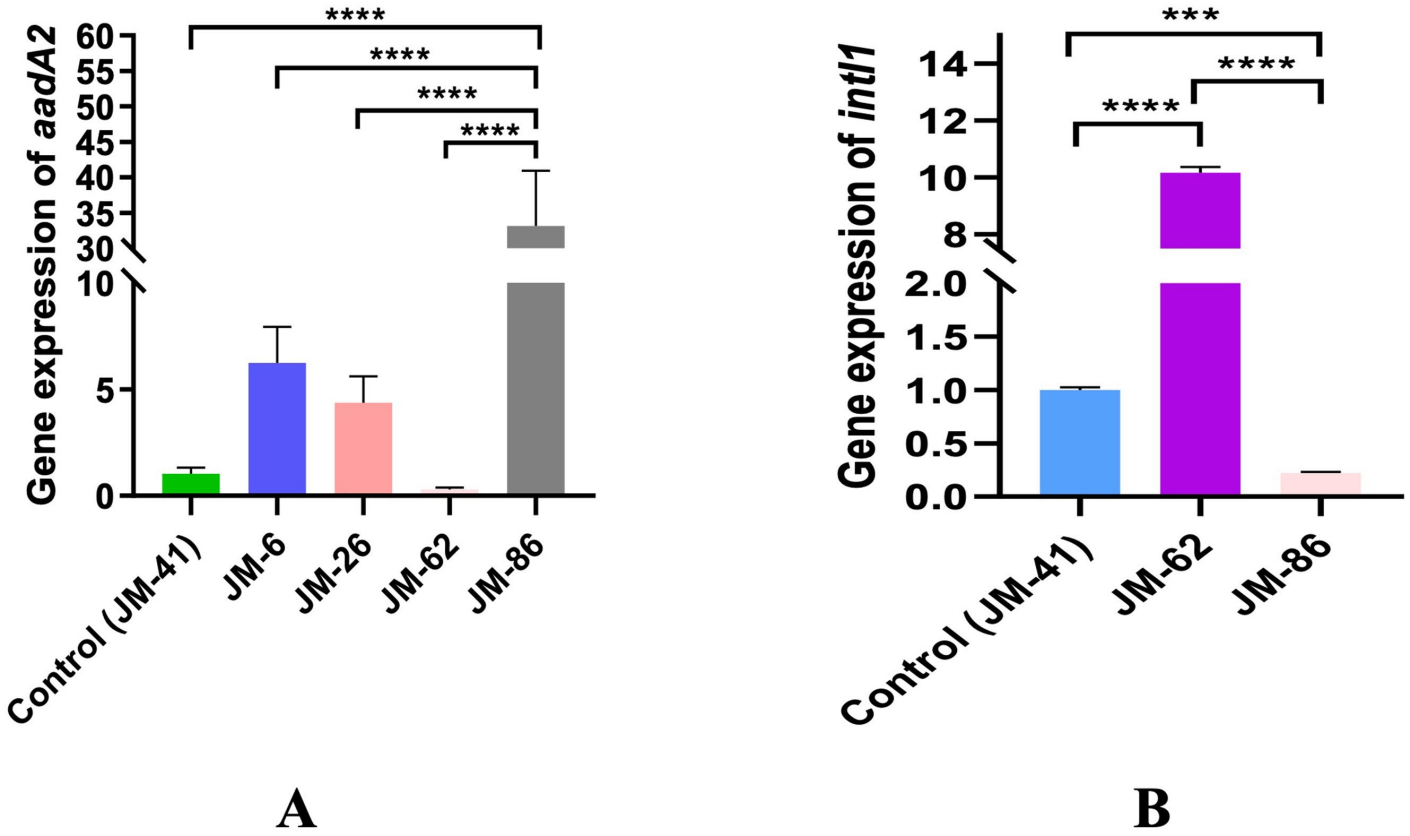
**(A)** The relative expression of *aadA2* in different constructed strains; **(B)** The relative expression of *intI1* in different constructed strains, ****p < 0.0001, ***p < 0.001.

The five constructed strains were divided into two groups based on their Pc promoter and *aadA2* gene cassette configurations: Group 1 comprised strains JM-41, JM-62, and JM-86, which had different Pc promoters but all carried the *aadA2* gene cassette. Group 2 included strains JM-6, JM-26, and JM-41, which had the same Pc promoter but varied in the distance between the *aadA2* gene cassette and the Pc promoter. In the qPCR analysis, strain JM-41 served as the reference strain. Multiple comparisons among the strains revealed that JM-86 exhibited the highest relative expression of the *aadA2* gene compared to the other four strains, with a significant statistical difference (*p* < 0.0001). The relative expression in JM-86 was approximately 100 times higher than that in JM-62, which had the lowest expression. The relative expression of the *intI1* gene followed the order JM-62 > JM-41 > JM-86, with statistically significant differences among the strains (*p* < 0.001). JM-62 showed approximately 50 times higher expression than JM-86, which had the lowest expression level. For further details, please refer to Figure 4.

### 3.6 Results of antimicrobial susceptibility testing for constructed strains

Antibiotic susceptibility testing was conducted on the five constructed strains carrying aminoglycoside and trimethoprim resistance gene cassettes using the microbroth dilution method for streptomycin, amikacin, chloramphenicol, and tetracycline. The results revealed streptomycin MIC values obtained by microbroth dilution were in the order JM-86 > JM-41 > JM-26 > JM-6 > JM-62. The K-B assay confirmed this trend, with inhibition zone diameters for streptomycin aligning closely with the microbroth dilution results (JM-86 < JM-41 < JM-26 < JM-6 = JM-62). Amikacin showed no variation in MIC values or inhibition zone diameters among the constructed strains, indicating susceptibility across all strains. All constructed strains exhibited resistance to chloramphenicol. Only JM-86 demonstrated intermediate resistance to tetracycline in both microbroth dilution and K-B assays, while the other strains were sensitive. The antibiotic sensitivity testing results for the quality control strain *E. coli* ATCC25922 and the negative control *E. coli* JM109 fell within the effective range, as shown in Table 4.

**Table 4:** The antimicrobial sensitivity test results of constructed strains to each antibiotic.

Strains\antibiotics	MIC (μg/ml)				K-B (mm)			
	STR	AK	CHL	TE	STR	AK	CHL	TE
JM-6	8	0.25	32	1	13	22	6	25
JM-26	16	0.25	32	1	11	23	6	26
JM-41	32	0.25	32	4	10	22	6	20
JM-62	4	0.25	32	4	13	21	6	23
JM-86	128	0.25	32	8	6	22	6	14
25922	4	0.5	2	1	20	23	27	23
JM109	1	0.25	4	2	15	21	25	26

STR, streptomycin; AK, amikacin; CHL, chloramphenicol; TE, tetracycline.

## 4 Discussion

*M. morganii*, the sole species within the genus *Morganella*, is a widely distributed bacterium that serves as a significant reservoir for the cloning and dissemination of various antibiotic resistance genes. In recent years, antibiotic resistance in *M. morganii* has been rapidly increasing, primarily driven by exogenous genetic elements such as transposons and integrons (Luo et al., 2022). While previous research on *M. morganii* has primarily focused on case analyses for treatment, epidemiology, and broad-spectrum *β*-lactamase analysis (Shrestha et al., 2020; Marado and Guerra, 2022; Alsaadi et al., 2024), studies investigating the correlation between its resistance mechanisms and the expression regulation of drug resistance gene cassettes within integrons are limited. This study employed clinical isolates of *M. morganii* positive for class 1 integrons collected over the past 6 years from our hospital. We initially screened these strains for drug resistance gene cassettes and promoters in the variable region, then analyzed the correlation between strain resistance and variable region promoters in integrons. Additionally, we constructed strains containing different types of promoters and gene cassettes in the variable region to conduct qPCR and antimicrobial susceptibility tests, thereby analyzing the regulatory role of variable region promoters in class 1 integrons on gene cassette expression.

Studies on class 1 integrons are more prevalent in *Enterobacteriaceae* bacteria such as *E. coli*, *Klebsiella pneumoniae*, and non-fermenting bacteria like *Pseudomonas aeruginosa* and *Acinetobacter baumannii* (Han et al., 2008; Wei et al., 2013; Bocharova et al., 2020; Farajzadeh Sheikh et al., 2024). In contrast, few studies have focused on *M. morganii* and class 1 integrons. Among the 97 non-duplicated clinical isolates of *M. morganii* in this study, the prevalence of class 1 integron-positive strains was 28.9%, lower than that observed in common *Enterobacteriaceae* bacteria. This lower prevalence might be attributed to the relatively lower detection rate of *M. morganii* in clinical settings. Wei et al. reported a detection rate of class 1 integrons in clinical isolates of *E. coli* as 72% (Wei et al., 2013), and in another study of *K. pneumoniae*, the positivity rate of class 1 integrons was 31.5% (Farajzadeh Sheikh et al., 2024). The host bacteria in these studies exhibited relatively higher levels of antibiotic resistance compared to the M. morganii isolates in the current study. The detected variable region gene cassettes in class 1 integron-positive strains primarily included aminoglycoside resistance genes (*aadA*, *aacA4*, *aadB*) and the trimethoprim resistance gene (*dfrA*), consistent with findings from other genera with class 1 integron-positive strains (Xiao et al., 2019; Li et al., 2022). In this study, some integron-positive strains failed to amplify gene cassettes, possibly due to atypical gene cassettes in the variable region. This could be attributed to gene recombination or insertion mutations in transposons, leading to the absence of the 3′ conserved segment or an excessive number of gene cassettes in the variable region. The latter may have exceeded the capability of conventional PCR amplification, resulting in amplification failure. Further validation using inverse PCR (Green and Sambrook, 2019; Figueroa-Bossi et al., 2024) techniques is necessary to address this issue (Odetoyin et al., 2017).

Extensive research has confirmed that promoters are crucial in regulating gene expression (Carrier et al., 2018; Brandis et al., 2021). Consequently, the expression of gene cassettes within integrons is significantly influenced by the variable region promoters. In this study, the predominant Pc promoter type in class 1 integron-positive strains was PcH1, a relatively weak promoter. The detection rates of PcS and PcW were lower than that of PcH1. The detection rate of PcH1 was also relatively high in other class 1 integron-positive Enterobacteriaceae. For instance, a study of class 1 integron-positive isolates of Proteus revealed a detection rate of PcH1 as high as 51%, making it the most common type (Xiao et al., 2019). Another study on the molecular characterization of class 1 integrons in carbapenem-resistant Enterobacteriaceae showed that PcH1 was also the predominant Pc (Wang et al., 2023). All downstream P2 promoters, containing 14 bases between the −35 and −10 regions, were inactive. Retrospective analysis of antibiotic resistance data in class 1 integron-positive strains did not reveal significant statistical differences in resistance rates to aminoglycosides or trimethoprim among strains with different Pc strengths. However, strains with stronger Pc promoters exhibited numerically higher resistance rates to these antibiotics than those with weaker Pc promoters. This observation reflects the fact that integrons contribute only partially to the host bacterium’s drug resistance genes. Furthermore, the expression and level of drug resistance genes may be influenced by various internal and external factors. Most integrons are located on plasmids or transposons (Liebert et al., 1999), which can potentially impact the resistance of the host bacterium, though not necessarily dominantly. To minimize interference and further explore the effect of variable region promoters within integrons on downstream gene expression, we used the low-copy plasmid pACYC184 as the vector. Recombinant plasmids were constructed to include variable region promoters and gene cassettes from class 1 integrons. Gene segments were synthesized based on actual sequencing results from experimental strains, preserving the natural configuration of integrons in these strains to a great extent. Additionally, HindIII and AseI were selected as the sites of enzyme digestion to replace the original *tet* promoter on the plasmid, positioning them away from the internal *cat* reference gene. This design allows the inserted gene segments to maximize the expression of drug resistance genes.

The strength of promoters and their distance from gene cassettes are primary factors influencing the transcription of downstream gene cassettes (Fonseca and Vicente, 2022). This project found significant differences in the expression efficiency of the antibiotic resistance gene cassette *aadA2* mediated by promoters of different strengths. qPCR results confirmed that stronger Pc promoters in integrons corresponded to higher transcription levels of downstream gene cassettes. In antimicrobial susceptibility tests, only strain JM-86, carrying a strong promoter, exhibited intermediate resistance to tetracycline in both microbroth dilution and K-B methods, whereas other strains remained sensitive. This suggests that the Pc promoter within the recombinant plasmid may regulate the downstream *tet* gene to some extent, highlighting the substantial impact of promoter strength on regulating gene expression. Interestingly, contrary to expectations based on other studies (Jacquier et al., 2009; Souque et al., 2021), the distance of Pc to downstream gene cassettes did not similarly affect gene expression in our experiment. Under the condition of identical Pc types, strains JM-6 and JM-26, located farther from Pc, exhibited higher relative expression levels of *aadA2* compared to the closer strain JM-41. Previous research in class 2 integrons has suggested that functional promoters of gene cassettes, like *ereA* located at the second position in the array, may enhance the expression of adjacent gene cassettes (Fonseca and Vicente, 2022), potentially explaining the increased expression of *aadA2* in strain JM-26 at a distance. Additionally, the copy number of plasmids within engineered bacteria also influences gene cassette expression levels (Stokes and Hall, 1989). Furthermore, the gene segments used in this study were derived from clinical strains containing unknown functional sequences within integrons that might also regulate the expression of gene cassettes in the variable region, warranting further investigation. Notably, the expression levels of *intI1* in strains with different Pc strengths exhibited an inverse relationship with *aadA2* expression: stronger Pc promoters correlated with higher expression levels of antibiotic resistance genes in the variable region but lower expression levels of *intI1*. This suggests that the integrase’s ability for integration and excision may decrease, maintaining bacterial stability internally, consistent with the literature (Wei et al., 2011). Differences in streptomycin MICs observed among engineered strains using microbroth dilution indicate varied regulatory effects of different promoter expressions, whereas no differences were observed for other aminoglycoside antibiotics like amikacin. This discrepancy likely stems from the product of *aadA2* being an aminoglycoside adenylyl transferase, which confers resistance to streptomycin and spectinomycin but not necessarily to other aminoglycosides (Bito and Susani, 1994; Walker et al., 2001).

The horizontal transfer of drug resistance genes is the fastest and most common way to spread clinical resistant strains (Warnes et al., 2012; Mathers et al., 2015), making homogeneity analysis of integron-positive strains particularly important. Herein, ERIC-PCR was used to analyze the homogeneity of 28 non-repetitive clinical isolates of *M. morganii* positive for class 1 integrons, using a similarity cutoff of 0.75. The results revealed three main genotypes: A, B, and C, comprising 11, 5, and 7 isolates, respectively. The distribution of promoter types and gene cassettes in the variable region was relatively concentrated within these genotypes. Furthermore, analysis of the strain data indicated that integron-positive *M. morganii* strains were predominantly found in the ICU and urology departments. This distribution is likely associated with the working environment of these departments. Factors such as surgical procedures in the ICU, critically ill patients, a high frequency of medical devices, and frequent operations increase the risk of cross-infection. Similarly, urological procedures involving catheterization and intravenous administration are invasive, contributing further to this risk. Therefore, it is crucial to enhance disinfection and sterilization of departmental environments and surgical instruments, as well as to standardize medical procedures such as intravenous administration and catheterization to minimize or prevent cross-infection. These measures aim to reduce the potential for clonal spread of integrons within hospitals.

Mobile genetic elements play a role in the horizontal transfer of drug resistance genes. Under antibiotic stress, class 1 integrons can capture gene cassette-like structures from DNA fragments ingested by bacteria and integrate them into attI1 sites through upregulation of integrase expression. Attempted transcription and translation of reading frames present in gene cassettes occur through variable region promoters and the mechanisms of translation termination-reinitiation coupling. Regardless of whether the reading frame in the gene cassette has a promoter or ribosomal binding site, if the expression product of the reading frame can confer resistance to the antibacterial agent, the strain can survive. The captured drug resistance gene cassettes can be disseminated through the proliferation and horizontal transfer of strains, leading to the emergence and dissemination of bacterial resistance (Guerin et al., 2009).

## 5 Conclusion

In summary, integron promoters exhibit a diverse distribution, predominantly carrying drug resistance gene cassettes for aminoglycosides and trimethoprim. The strength of the class 1 integron variable region promoters significantly correlates with the expression levels of downstream gene cassettes. It is likely that the clonal spread of class 1 integron-positive *M. morganii* occurs within our local healthcare setting. This study aimed to investigate the promoter distribution characteristics of class 1 integrons and their regulatory effects on drug resistance genes in *M. morganii* isolates, providing a theoretical basis for the prevention and treatment of infections with *M. morganii* in clinical practice. Additionally, this study may offer new insights for addressing other rare clinical bacteria with increasing drug resistance in the future. The effect of antibiotic factors on integrons will be studied in our future work.

## Data Availability

The datasets presented in this study can be found in online repositories. The names of the repository/repositories and accession number(s) can be found in the article/Supplementary material.

## References

[ref1] Agrawal K. U. Limaye Joshi K. Gad M. (2021). A rare case of fulminant acute postoperative *Morganella morganii* Endophthalmitis. Ocul. Immunol. Inflamm. 31, 123–126. doi: 10.1080/09273948.2021.1993269, PMID: 34802374

[ref2] Alelyani F. M. Almutawif Y. A. Ali H. M. Aljohani R. Z. Almutairi A. Z. Murshid W. R. (2022). A pituitary abscess caused by *Morganella morganii*: a case report. Am. J. Case Rep. 23:e936743. doi: 10.12659/ajcr.936743, PMID: 36110038 PMC9486448

[ref3] Alsaadi A. Alghamdi A. A. Akkielah L. Alanazi M. Alghamdi S. Abanamy H.. (2024). Epidemiology and clinical characteristics of *Morganella morganii* infections: a multicenter retrospective study. J. Infect. Public Health 17, 430–434. doi: 10.1016/j.jiph.2023.12.013, PMID: 38262080

[ref4] Bakhshi B. Afshari N. Fallah F. (2018). Enterobacterial repetitive intergenic consensus (ERIC)-PCR analysis as a reliable evidence for suspected *Shigella* spp. outbreaks. Braz. J. Microbiol. 49, 529–533. doi: 10.1016/j.bjm.2017.01.014, PMID: 29482996 PMC6066780

[ref5] Banoub J. Kundu J. Kansal S. Rathore S. Kaundal M. Angrup A.. (2022). Evaluation of ERIC-PCR and MALDI-TOF as typing tools for multidrug resistant *Klebsiella pneumoniae* clinical isolates from a tertiary care center in India. PLoS One 17:e0271652. doi: 10.1371/journal.pone.0271652, PMID: 36395172 PMC9671336

[ref6] Behera D. U. Dixit S. Gaur M. Mishra R. Sahoo R. K. Sahoo M.. (2023a). Sequencing and characterization of *M. morganii* strain UM869: a comprehensive comparative genomic analysis of virulence, antibiotic resistance, and functional pathways. Genes 14:1279. doi: 10.3390/genes14061279, PMID: 37372459 PMC10298637

[ref7] Behera D. U. Ratnajothy K. Dey S. Gaur M. Sahoo R. K. Sahoo S.. (2023b). *In vitro* synergistic interaction of colistin and other antimicrobials against intrinsic colistin-resistant *Morganella morganii* isolates. 3 Biotech 13:127. doi: 10.1007/s13205-023-03551-w, PMID: 37064006 PMC10097849

[ref8] Bito A. Susani M. (1994). Revised analysis of aadA2 gene of plasmid pSa. Antimicrob. Agents Chemother. 38, 1172–1175. doi: 10.1128/aac.38.5.1172, PMID: 7915099 PMC188172

[ref9] Bocharova Y. Savinova T. Lazareva A. Polikarpova S. Gordinskaya N. Mayanskiy N.. (2020). Genotypes, carbapenemase carriage, integron diversity and oprD alterations among carbapenem-resistant *Pseudomonas aeruginosa* from Russia. Int. J. Antimicrob. Agents 55:105899. doi: 10.1016/j.ijantimicag.2020.105899, PMID: 31931151

[ref10] Brandis G. Gockel J. Garoff L. Guy L. Hughes D. (2021). Expression of the qepA1 gene is induced under antibiotic exposure. J. Antimicrob. Chemother. 76, 1433–1440. doi: 10.1093/jac/dkab045, PMID: 33608713 PMC8120332

[ref11] Carrier M.-C. Lalaouna D. Massé E. (2018). Broadening the definition of bacterial small RNAs: characteristics and mechanisms of action. Ann. Rev. Microbiol. 72, 141–161. doi: 10.1146/annurev-micro-090817-062607, PMID: 30200848

[ref12] CLSI (2022). Performance standards for antimicrobial susceptibility testing. 32nd Edn CLSI supplement M100. Wayne, PA: Clinical and Laboratory Standards Institute.

[ref13] Coluzzi C. Guillemet M. Mazzamurro F. Touchon M. Godfroid M. Achaz G.. (2023). Chance favors the prepared Genomes_ horizontal transfer shapes the emergence of antibiotic resistance mutations in Core genes. Mol. Biol. Evol. 40:msad217. doi: 10.1093/molbev/msad217, PMID: 37788575 PMC10575684

[ref14] Elmi S. M. Obame F. L. O. Dokponou Y. C. H. Yassin M. R. Attari S. E. El Asri A. C. C.. (2024). Brain abscess caused by *Morganella morganii*: a case report and review of the literature. Surg. Neurol. Int. 15:7. doi: 10.25259/sni_759_2023, PMID: 38344080 PMC10858772

[ref15] Farajzadeh Sheikh A. Abdi M. Farshadzadeh Z. (2024). Molecular detection of class 1, 2, and 3 integrons in hypervirulent and classic *Klebsiella pneumoniae* isolates: a cross-sectional study. Health Sci. Rep. 7:e1962. doi: 10.1002/hsr2.1962, PMID: 38698788 PMC11063457

[ref16] Figueroa-Bossi N. Balbontín R. Bossi L. (2024). Mapping transposon insertion sites by inverse polymerase chain reaction and sanger sequencing. Cold Spring Harb. Protoc. 2024:108197. doi: 10.1101/pdb.prot108197, PMID: 37188521

[ref17] Fonseca É. L. Vicente A. C. (2022). Integron functionality and genome innovation: an update on the subtle and smart strategy of integrase and gene cassette expression regulation. Microorganisms 10:224. doi: 10.3390/microorganisms10020224, PMID: 35208680 PMC8876359

[ref18] Gameiro I. Botelho T. Martins A. I. Henriques R. Lapa P. (2023). *Morganella morganii*: a rare cause of early-onset neonatal Sepsis. Cureus 15:e45600. doi: 10.7759/cureus.45600, PMID: 37868540 PMC10588523

[ref19] Green M. R. Sambrook J. (2019). Inverse polymerase chain reaction (PCR). Cold Spring Harbor Protoc. 2019, 170–174. doi: 10.1101/pdb.prot095166 30710023

[ref20] Guerin E. Cambray G. Sanchez-Alberola N. Campoy S. Erill I. Da Re S.. (2009). The SOS response controls integron recombination. Science 324:1034. doi: 10.1126/science.1172914, PMID: 19460999

[ref21] Hall R. M. Collis C. M. (2006). Mobile gene cassettes and integrons: capture and spread of genes by site-specific recombination. Mol. Microbiol. 15, 593–600. doi: 10.1111/j.1365-2958.1995.tb02368.x, PMID: 7783631

[ref22] Hall R. M. Stokes H. W. (1993). Integrons:novel DNA elements which capture genes by site-specific recombination. Genetica 90, 115–132. doi: 10.1007/BF01435034, PMID: 8119588

[ref23] Han H. L. Jang S. J. Park G. Kook J. K. Shin J. H. Shin S. H.. (2008). Identification of an atypical integron carrying an IS26-disrupted aadA1 gene cassette in *Acinetobacter baumannii*. Int. J. Antimicrob. Agents 32, 165–169. doi: 10.1016/j.ijantimicag.2008.03.009, PMID: 18565738

[ref24] Hanau-Berçot B. Podglajen I. Casin I. Collatz E. (2002). An intrinsic control element for translational initiation in class 1 integrons. Mol. Microbiol. 44, 119–130. doi: 10.1046/j.1365-2958.2002.02843.x, PMID: 11967073

[ref25] Jacquier H. Zaoui C. Sanson-Le Pors M. J. Mazel D. Berçot B. (2009). Translation regulation of integrons gene cassette expression by the attC sites. Mol. Microbiol. 72, 1475–1486. doi: 10.1111/j.1365-2958.2009.06736.x, PMID: 19486293

[ref26] Kvopka M. Chan W. Baranage D. Sia D. (2023). Morganella morganii and *Enterococcus faecalis* endophthalmitis following intravitreal injection. BMC Ophthalmol. 23:450. doi: 10.1186/s12886-023-03198-4, PMID: 37950172 PMC10638747

[ref27] Laupland K. Paterson D. Edwards F. Stewart A. Harris P. (2022). *Morganella morganii*, an emerging cause of bloodstream infections. Microbiol. Spectr. 10:e0056922. doi: 10.1128/spectrum.00569-22, PMID: 35467403 PMC9241912

[ref28] Leverstein-Van Hall M. Box A. Blok H. Paauw A. Fluit A. Verhoef J. (2002). Evidence of extensive interspecies transfer of integron-mediated antimicrobial resistance genes among multidrug-resistant Enterobacteriaceae in a clinical setting. J. Infect. Dis. 186, 49–56. doi: 10.1086/341078, PMID: 12089661

[ref29] Li W. Ma J. Sun X. Liu M. Wang H. (2022). Antimicrobial resistance and molecular characterization of gene cassettes from class 1 Integrons in *Escherichia coli* strains. Microb. Drug Resist. 28, 413–418. doi: 10.1089/mdr.2021.0172, PMID: 35076316 PMC9058876

[ref30] Li C. Wang H. Zhang J. Wang Z. Wei Y. Zhu Y. (2023). Endocarditis induced by *M. morganii* in an immunocompetent patient without underlying valvular abnormalities. Heliyon 9:e17069. doi: 10.1016/j.heliyon.2023.e17069, PMID: 37484222 PMC10361220

[ref31] Liebert C. A. Hall R. M. Summers A. O. (1999). Transposon Tn 21, flagship of the floating genome. Microbiol. Mol. Biol. Rev. 63, 507–522. doi: 10.1128/mmbr.63.3.507-522.1999, PMID: 10477306 PMC103744

[ref32] Liu H. Zhu J. Hu Q. Rao X. (2016). *Morganella morganii*, a non-negligent opportunistic pathogen. Int. J. Infect. Dis. 50, 10–17. doi: 10.1016/j.ijid.2016.07.006, PMID: 27421818

[ref33] Lu W. Qiu X. Chen K. Zhao R. Li Q. Wu Q. (2022). Distribution and molecular characterization of functional class 2 Integrons in clinical *Proteus mirabilis* isolates. Infect. Drug Resist. 15, 465–474. doi: 10.2147/idr.S347119, PMID: 35210790 PMC8858760

[ref34] Luo X. W. Liu P. Y. Miao Q. Q. Han R. J. Wu H. Liu J. H.. (2022). Multidrug resistance genes carried by a novel transposon Tn7376 and a Genomic Island named MMGI-4 in a pathogenic *Morganella morganii* isolate. Microbiol. Spectr. 10:e0026522. doi: 10.1128/spectrum.00265-22, PMID: 35510850 PMC9241818

[ref35] Marado D. Guerra M. (2022). Fulminans Purpura due to *Morganella morganii*. Eur. J. Case Rep. Internal Med. 9:003670. doi: 10.12890/2022_003670, PMID: 36506741 PMC9728211

[ref36] Mathers A. J. Peirano G. Pitout J. D. D. (2015). The role of epidemic resistance plasmids and international high-risk clones in the spread of multidrug-resistant Enterobacteriaceae. Clin. Microbiol. Rev. 28, 565–591. doi: 10.1128/cmr.00116-14, PMID: 25926236 PMC4405625

[ref37] Meacham K. J. Zhang L. Foxman B. Bauer R. J. Marrs C. F. (2003). Evaluation of genotyping large numbers of *Escherichia coli* isolates by Enterobacterial repetitive intergenic consensus-PCR. J. Clin. Microbiol. 41, 5224–5226. doi: 10.1128/jcm.41.11.5224-5226.2003, PMID: 14605168 PMC262489

[ref38] Novačić A. Menéndez D. Ljubas J. Barbarić S. Stutz F. Soudet J.. (2022). Antisense non-coding transcription represses the PHO5 model gene at the level of promoter chromatin structure. PLoS Genet. 18:e1010432. doi: 10.1371/journal.pgen.1010432, PMID: 36215302 PMC9584416

[ref39] Odetoyin B. W. Labar A. S. Lamikanra A. Aboderin A. O. Okeke I. N. (2017). Classes 1 and 2 integrons in faecal *Escherichia coli* strains isolated from mother-child pairs in Nigeria. PLoS One 12:e0183383. doi: 10.1371/journal.pone.0183383, PMID: 28829804 PMC5568733

[ref41] Shi H. Chen X. Yao Y. Xu J. (2022). *Morganella morganii*: an unusual analysis of 11 cases of pediatric urinary tract infections. J. Clin. Lab. Anal. 36:e24399. doi: 10.1002/jcla.24399, PMID: 35349730 PMC9102756

[ref42] Shrestha S. Tada T. Sherchan J. B. Uchida H. Hishinuma T. Oshiro S.. (2020). Highly multidrug-resistant *Morganella morganii* clinical isolates from Nepal co-producing NDM-type metallo-β-lactamases and the 16S rRNA methylase ArmA. J. Med. Microbiol. 69, 572–575. doi: 10.1099/jmm.0.001160, PMID: 32100711

[ref43] Souque C. Escudero J. A. Maclean R. C. (2021). Integron activity accelerates the evolution of antibiotic resistance. eLife 10:e62474. doi: 10.7554/eLife.62474 33634790 PMC8024014

[ref44] Stokes H. W. Hall R. M. (1989). A novel family of potentially mobile DNA elements encoding site-specific gene-integration functions: integrons. Mol. Microbiol. 3, 1669–1683. doi: 10.1111/j.1365-2958.1989.tb00153.x, PMID: 2560119

[ref45] Stokes H. W. O'Gorman D. B. Recchia G. D. Parsekhian M. Hall R. M. (1997). Structure and function of 59-base element recombination sites associated with mobile gene cassettes. Mol. Microbiol. 26, 731–745. doi: 10.1046/j.1365-2958.1997.6091980.x, PMID: 9427403

[ref46] Tseng C.-S. Yen Y.-C. Chang C.-C. Hsu Y.-M. (2014). Polymorphism of gene cassette promoter variants of class 1 integron harbored in S. Choleraesuis and typhimurium isolated from Taiwan. Biomedicine 4:20. doi: 10.7603/s40681-014-0020-3, PMID: 25520933 PMC4264977

[ref47] Von Wintersdorff C. J. H. Penders J. Van Niekerk J. M. Mills N. D. Majumder S. Van Alphen L. B.. (2016). Dissemination of antimicrobial resistance in microbial ecosystems through horizontal gene transfer. Front. Microbiol. 7:173. doi: 10.3389/fmicb.2016.00173, PMID: 26925045 PMC4759269

[ref48] Wang T. Zhu Y. Zhu W. Cao M. Wei Q. (2023). Molecular characterization of class 1 integrons in carbapenem-resistant Enterobacterales isolates. Microb. Pathog. 177:106051. doi: 10.1016/j.micpath.2023.106051, PMID: 36858185

[ref40] Walker R. A. Lindsay E. Woodward M. J. Ward L. R. Threlfall E. J. (2001). Variation in clonality and antibiotic-resistance genes among multiresistant *Salmonella enterica* serotype typhimurium phage-type U302 (MR U302) from humans, animals, and foods. Microb. Drug Resist. 7, 13–21. doi: 10.1089/107662901750152701, PMID: 11310799

[ref49] Warnes S. L. Highmore C. J. Keevil C. W. (2012). Horizontal transfer of antibiotic resistance genes on abiotic touch surfaces: implications for public health. MBio 3, e00489–e00412. doi: 10.1128/mBio.00489-12 23188508 PMC3509412

[ref50] Wei Q. H. (2010). The research of regulation mechamism for integron capturing and expressing 477 antibiotic resistance gene cassettes in bacteria. Ph.D. Thesis,: Fudan University.

[ref51] Wei Q. Jiang X. Li M. Chen X. Li G. Li R.. (2011). Transcription of integron-harboured gene cassette impacts integration efficiency in class 1 integron. Mol. Microbiol. 80, 1326–1336. doi: 10.1111/j.1365-2958.2011.07648.x, PMID: 21453444

[ref52] Wei Q. Jiang X. Li M. Li G. Hu Q. Lu H.. (2013). Diversity of gene cassette promoter variants of class 1 Integrons in Uropathogenic *Escherichia coli*. Curr. Microbiol. 67, 543–549. doi: 10.1007/s00284-013-0399-1, PMID: 23743598

[ref53] Xiao L. Wang X. Kong N. Cao M. Zhang L. Wei Q.. (2019). Polymorphisms of gene cassette promoters of the class 1 Integron in clinical Proteus isolates. Front. Microbiol. 10:790. doi: 10.3389/fmicb.2019.00790, PMID: 31068909 PMC6491665

[ref54] Yeşil M. Özcan Ö. Karasu N. Kağan Yılmaz B. (2023). Atypical compartment syndrome of the forearm due to mixed infection with Proteus mirabilis and *Morganella morganii* after a penetrating injury: a limb-saving approach. Joint Dis. Relat. Surg. 34, 752–756. doi: 10.52312/jdrs.2023.1066, PMID: 37750284 PMC10546849

[ref55] Zaric R. Z. Jankovic S. Zaric M. Milosavljevic M. Stojadinovic M. Pejcic A. (2021). Antimicrobial treatment of *Morganella morganii* invasive infections: systematic review. Indian J. Med. Microbiol. 39, 404–412. doi: 10.1016/j.ijmmb.2021.06.005, PMID: 34193353

