# Supplementary material for: Distribution diversity and expression regulation of class 1 integron promoters in clinical isolates of Morganella morganii
Source: Front Microbiol. 2024 Oct 18;15:1459162. doi: 10.3389/fmicb.2024.1459162 (PMC11527653; doi:10.3389/fmicb.2024.1459162)
Supplement: Supplementary file 4 [file Data_Sheet_4.DOCX]

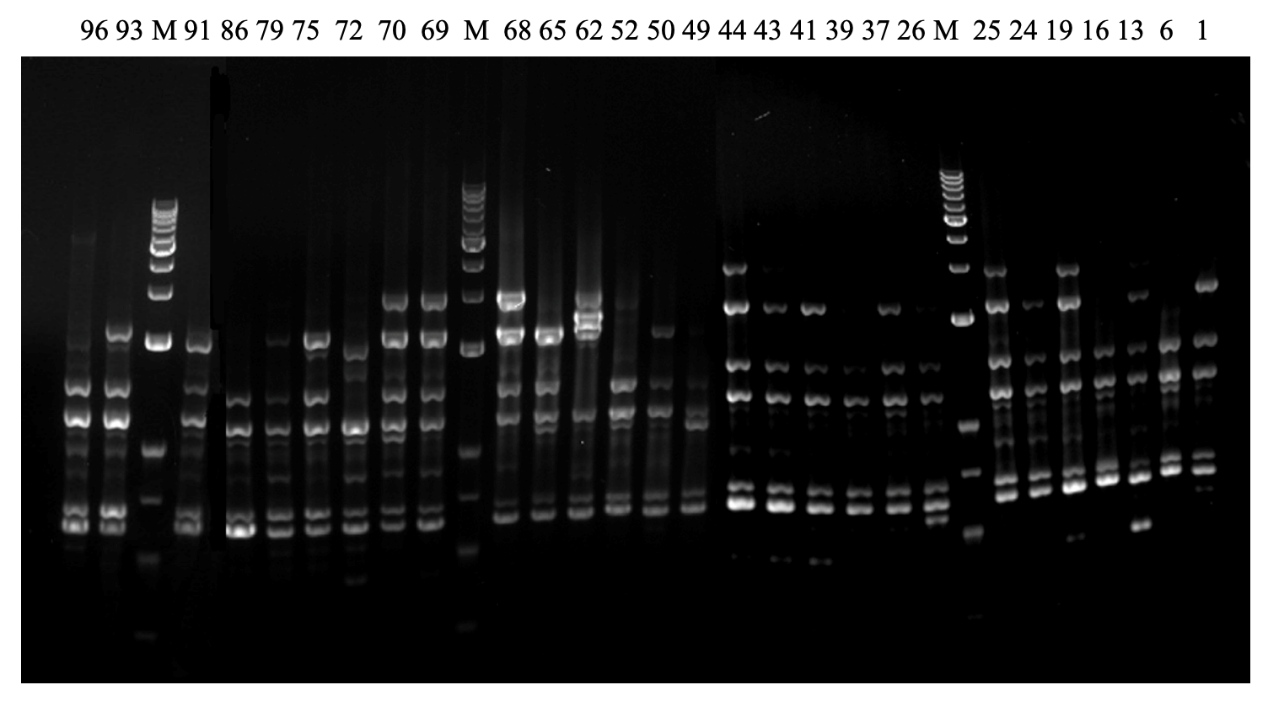


Supplementary Fig1. ERIC-PCR electrophoretogram. The negative pole of each lane was marked with the strain number. M: marker
