# Supplementary material for: Distribution diversity and expression regulation of class 1 integron promoters in clinical isolates of Morganella morganii
Source: Front Microbiol. 2024 Oct 18;15:1459162. doi: 10.3389/fmicb.2024.1459162 (PMC11527653; doi:10.3389/fmicb.2024.1459162)
Supplement: Supplementary file 5 [file Data_Sheet_5.DOCX]

JM-6

ATTAATctacctctcactagtgaggggcggcagcgcatcaagcggtgagcgcactccggcaccgccaactttcagcacatgcgtgtaaatcatcgtcgtagagacgtcggaatggccgagcagatcctgcacggttcgaatgtcgtaaccgctgcggagcaaggccgtcgcgaacgagtggcggagggtgtgcggtgtggcgggcttcgtgatgcctgcttgttctacggcacgtttgaaggcgcgctgaaaggtctggtcatacatgtgatggcgacgcacgacaccgctccgtggatcggtcgaatgcgtgtgctgcgcaaaaacccagaaccacggccaggaatgcccggcgcgcggatacttccgctcaagggcgtcgggaagcgcaacgccgctgcggccctcggcctggtccttcagccaccatgcccgtgcacgcgacagctgctcgcgcaggctgggtgccaagctctcgggtaacatcaaggcccgatccttggagcccttgccctcccgcacgatgatcgtgccgtgatcgaaatccagatccttgacccgcagttgcaaaccctcactgatccgcatgcccgttccatacagaagctgggcgaacaaacgatgctcgccttccagaaaaccgaggatgcgaaccacttcatccggggtcagcaccaccggcaagcgccgcgacggccgaggtcttccgatctcctgaagccagggcagatccgtgcacagcaccttgccgtagaagaacagcaaggccgccaatgcctgacgatgcgtggagaccgaaaccttgcgctcgttcgccagccaggacagaaatgcctcgacttcgctgctgcccaaggttgccgggtgacgcacaccgtggaaacggatgaaggcacgaacccagtggacataagcctgttcggttcgtaaactgtaatgcaagtagcgtatgcgctcacgcaactggtccagaac

intI1

-35 -10

Pc promoter

cttgaccgaacgcagcggtggtaacggcgcagtggcggttttcatggcttgttatgactgtttttttgtacagtctatgcctcgggcatccaagcagcaagcgcgttacgccgtgggtcgatgtttgatgttatggagcagcaacgatgttacgcagcagggcagtcgccctaaaacaaagttagccatatgaactcggaatcagtacgcatttatctcgttgctgcgatgggagccaatcgggttattggcaatggtcctaatatcccctggaaaattccgggtgagcagaagatttttcgcagactcactgagggaaaagtcgttgtcatggggcgaaagacctttgagtctatcggcaagcctctaccgaaccgtcacacattggtaatctcacgccaagctaactaccgcgccactggctgcgtagttgtttcaacgctgtcgcacgctatcgctttggcatccgaactcggcaatgaactctacgtcgcgggcggagctgagatatacactctggcactacctcacgcccacggcgtgtttctatctgaggtacatcaaaccttcgagggtgacgccttcttcccaatgctcaacgaaacagaattcgagcttgtctcaaccgaaaccattcaagctgtaattccgtacacccactccgtttatgcgcgtcgaaacggctaaccattccgtcaacgggacgccaaaatgctgcgcattttggttccctccgctgcgctccggctctcgttacgtccaacgttagcaccactgaaacccagctttatttagctcatgtttattcaaacggcatttagcttttcaggcgttattcagtgcctgttttgccttttttccgggcttcgcctgcatgggctgcgcaggttttcagtctttttggcctctagcccttgcgtagcaagcgcaagcagctatcgtttttgcagtgctgtgccgcctcggtggcgcagcgttttttcacggttagcgcccgtcgccaaattcaagttatccgttttggcttctggttctaacatttcggtcaagccgacccgcattctgcggtcggcttacctcgcccgttagacatcatgagggaagcggtgaccatcgaaatttcgaaccaactatcagaggtgctaagcgtcattgagcgccatctggaatcaacgttgctggccgtgcatttgtacggctccgcagtggatggcggcctgaagccatacagcgatattgatttgttggttactgtggccgtaaagcttgatgaaacgacgcggcgagcattgctcaatgatcttatggaggcttcggctttccctggcgagagcgagacgctccgcgctatagaagtcacccttgtcgtgcatgacgacatcatcccgtggcgttatccggctaagcgcgagctgcaatttggagaatggcagcgcaatgacattcttgcgggtatcttcgagccagccatgatcgacattgatctagctatcctgcttacaaaagcaagagaacatagcgttgccttggtaggtccggcagcggaggaattctttgacccggttcctgaacaggatctattcgaggcgctgagggaaaccttgaagctatggaactcgcagcccgactgggccggcgatgagcgaaatgtagtgcttacgttgtcccgcatttggtacagcgcaataaccggcaaaatcgcgccgaaggatgtcgctgccgactgggcaataaaacgcctacctgcccagtatcagcccgtcttacttgaagctaagcaagcttatctgggacaaaaagaagatcacttggcctcacgcgcagatcacttggaagaatttattcgctttgtgaaaggcgagatcatcaagtcagttggtaaatgatgtctaacaattcgttcaagccgaccgcgctacgcgcggcggcttaactccggcgttagatgcactaagcacataatgctcacagccaaagctaaaAAGCTT

aadA2

attc1

dfrA12

JM-26

ATTAATctacctctcactagtgaggggcggcagcgcatcaagcggtgagcgcactccggcaccgccaactttcagcacatgcgtgtaaatcatcgtcgtagagacgtcggaatggccgagcagatcctgcacggttcgaatgtcgtaaccgctgcggagcaaggccgtcgcgaacgagtggcggagggtgtgcggtgtggcgggcttcgtgatgcctgcttgttctacggcacgtttgaaggcgcgctgaaaggtctggtcatacatgtgatggcgacgcacgacaccgctccgtggatcggtcgaatgcgtgtgctgcgcaaaaacccagaaccacggccaggaatgcccggcgcgcggatacttccgctcaagggcgtcgggaagcgcaacgccgctgcggccctcggcctggtccttcagccaccatgcccgtgcacgcgacagctgctcgcgcaggctgggtgccaagctctcgggtaacatcaaggcccgatccttggagcccttgccctcccgcacgatgatcgtgccgtgatcgaaatccagatccttgacccgcagttgcaaaccctcactgatccgcatgcccgttccatacagaagctgggcgaacaaacgatgctcgccttccagaaaaccgaggatgcgaaccacttcatccggggtcagcaccaccggcaagcgccgcgacggccgaggtcttccgatctcctgaagccagggcagatccgtgcacagcaccttgccgtagaagaacagcaaggccgccaatgcctgacgatgcgtggagaccgaaaccttgcgctcgttcgccagccaggacagaaatgcctcgacttcgctgctgcccaaggttgccgggtgacgcacaccgtggaaacggatgaaggcacgaacccagtggacataagcctgttcggttcgtaaactgtaatgcaagtagcgtatgcgctcacgcaactggtccagaac

dfrA32

intI1

ere(A)1

-35 -10

Pc promoter

cttgaccgaacgcagcggtggtaacggcgcagtggcggttttcatggcttgttatgactgtttttttgtacagtctatgcctcgggcatccaagcagcaagcgcgttacgccgtgggtcgatgtttgatgttatggagcagcaacgatgttacgcagcagggcagtcgccctaaaacaaagttagccatatcgggagttaaattgaaaatttcattgatttctgcagtgtcagaaaatggcgtaatcggtagtggtcctgatattccgtggtcagcaaaaggtgagcagctaatctttaaggcgctcacatacaatcagtggcttcttgttggaaggaaaacatttgactctatgggagttcttccaaatcgcaaatatgcagtagtgtcaaagaatggaatttcagggtcaaatgaaaacgtcttggtttttccttcaatagaaaatgctttgcaagaactatctaaaattacagatcatgtatatatttcgggtggggggcaaatctatgaaagccttattgaaaaagcagatataattcatctatctactattcatgttgaggttgaaggtgatattaaattccctatattacctgaaggtttcaacttggtttttgaacagttttttgtgtctaatataaattatacatatcaaatttggaaaaaaggctaacaagtcgttgcagcaccagtcgctccgctccttggacagtttttaagttgtggttttatggttttgctgcgcaaaaatattccataaaaccacaacttaaaaactgccgctgaactcggcgttatgctgtgacccgggttgttggcgaagcgaacgtatggcgattaaagtaataaacgcaaaggtaaaataatgacgtggagaacgaccagaacacttttacagcctcaaaatctggacttcaatgagtttgagattcttacttccgtaattgagggcgcccgaattgtcggcattggcgagggcgctcattttgtcgcggagttttcactggctagagcaagtcttatccgctatttggtcgaaaggcatgattttaatgcgattggtttggaatgtggggcgattcaggcatcccggttatctgaatggctcaactcaacagccggtgctcatgaacttgagcgattttcggataccctgaccttttctgtgtatggctcagtgctgatctggctgaaatcatatctccgcgaatcaggaagaaaactgcagttagtcggaatcgacttacccaacaccctgaacccaagggacgacctagcgcaattggccgaaattatccagctcatcgatcacctcatgaaaccgcacgttgatatgctgactcacttgttggcgtccattgatggccagtcggcggttatttcatcggcaaaatggggggagctagaaacggctcggcaggagaaagctatctcaggggtaaccagattgaagctccgcttggcgtcgcttgcccctgtactgaaaaaacacgtcaacagcgatttgttccgaaaagcctctgatcgaatagaatcgatagagtatacgttggaaaccttgcgtataatgaaaactttcttcgatggtacctctcttgagggagatacttccgtacgtgactcgtatatggcgggcgtagtagatggaatggttcgagcgaatccggatgtgaagataattctgctggcgcacaacaatcatttacaaaaaaccccagtctccttttcaggcgagcttacggctgttcccatggggcagcacctcgcagagagggtgaattaccgtgcgattgcattcacccatcttggacccaccgtgccggaaatgcatttcccatcgcccgacagtcctcttggattctctgttgtgaccacgcctgccgatgcaatccgtgaggatagtatggaacagtatgtcatcgacgcctgtggtacggagaattcatgtctgacattgacagatgcccccatggaagcaaagcgaatgcggtctcaaagcgcctctgtaaaaacgaaattgagcgaggcatttgatgccatcgtctgtgttccaagcgccggcaaggacagcctagttgccctataggaaaccggaaatgaaaatgagggagcataacctgcgaatccaccggacggttttcaaccgccggtgatcagcgcgttagacatcatgagggtagcggtgaccatcgaaatttcgaaccaactatcagaggtgctaagcgtcattgagcgccatctggaatcaacgttgctggccgtgcatttgtacggctccgcagtggatggcggcctgaagccatacagcgatattgatttgttggttactgtggccgtaaagcttgatgaaacgacgcggcgagcattgctcaatgaccttatggaggcttcggctttccctggcgagagcgagacgctccgcgctatagaagtcacccttgtcgtgcatgacgacatcatcccgtggcgttatccggctaagcgcgagctgcaatttggagaatggcagcgcaatgacattcttgcgggtatcttcgagccagccatgatcgacattgatctagctatcctgcttacaaaagcaagagaacatagcgttgccttggtaggtccggcagcggaggaattctttgacccggttcctgaacaggatctattcgaggcgctgagggaaaccttgaagctatggaactcgcagcccgactgggccggcgatgagcgaaatgtagtgcttacgttgtcccgcatttggtacagcgcaataaccggcaaaatcgcgccgaaggatgtcgctgccgactgggcaataaaacgcctacctgcccagtatcagcccgtcttacttgaagctaagcaagcttatctgggacaaaaagaagatcacttggcctcacgcgcagatcacttggaagaatttattcgctttgtgaaaggcgagatcatcaagtcagttggtaaatgatgtctaacaattcgttcaagccgaccgcgctacgcgcggcggcttaactccggcgttagatgcactaagcacataattgctcacagccaaagccttatgatgttacccgagagcttggtgatgttaaccccagagcttggcacccagcctgcgcgagcagctgtcgcgtgcacgggcatggtggctgaaggaccaggccgagggccgcagcggcgttgcgcttcccgacgcccttgagcggaagtatccgcgcgccgggcattcctggccgtggttctgggtttttgcgcagcacacgcattcgaccgatccacggagcggtgtcgtgcgtcgccatcacatgtatgaccagacctttcagcgcgccttcaaacgtgccgtagaacaagcaggcatcacgaagcccgccacaccgcacaccctccgccactcgttcgcgacggccttgctccgcagcggttacgacattcgaaccgtgcaggatctgctcggccattccgacgtctctacgacgatgatttacacgcatgtgctgaaagttggcggtgccggagtgcgctcaccgcttgatgcgctgccgcccctcactagtgagaggtagAAGCTT

attc1

attc2

aadA2

JM-41

ATTAATctacctctcactagtgaggggcggcagcgcatcaagcggtgagcgcactccggcaccgccaactttcagcacatgcgtgtaaatcatcgtcgtagagacgtcggaatggccgagcagatcctgcacggttcgaatgtcgtaaccgctgcggagcaaggccgtcgcgaacgagtggcggagggtgtgcggtgtggcgggcttcgtgatgcctgcttgttctacggcacgtttgaaggcgcgctgaaaggtctggtcatacatgtgatggcgacgcacgacaccgctccgtggatcggtcgaatgcgtgtgctgcgcaaaaacccagaaccacggccaggaatgcccggcgcgcggatacttccgctcaagggcgtcgggaagcgcaacgccgctgcggccctcggcctggtccttcagccaccatgcccgtgcacgcgacagctgctcgcgcaggctgggtgccaagctctcgggtaacatcaaggcccgatccttggagcccttgccctcccgcacgatgatcgtgccgtgatcgaaatccagatccttgacccgcagttgcaaaccctcactgatccgcatgcccgttccatacagaagctgggcgaacaaacgatgctcgccttccagaaaaccgaggatgcgaaccacttcatccggggtcagcaccaccggcaagcgccgcgacggccgaggtcttccgatctcctgaagccagggcagatccgtgcacagcaccttgccgtagaagaacagcaaggccgccaatgcctgacgatgcgtggagaccgaaaccttgcgctcgttcgccagccaggacagaaatgcctcgacttcgctgctgcccaaggttgccgggtgacgcacaccgtggaaacggatgaaggcacgaacccagtggacataagcctgttcggttcgtaaactgtaatgcaagtagcgtatgcgctcacgcaactggtccagaac

intI1

-35 -10

Pc promoter

cttgaccgaacgcagcggtggtaacggcgcagtggcggttttcatggcttgttatgactgtttttttgtacagtctatgcctcgggcatccaagcagcaagcgcgttacgccgtgggtcgatgtttgatgttatggagcagcaacgatgttacgcagcagggcagtcgccctaaaacaaagttagacatcatgagggaagcggtgaccatcgaaatttcgaaccaactatcagaggtgctaagcgtcattgagcgccatctggaatcaacgttgctggccgtgcatttgtacggctccgcagtggatggcggcctgaagccatacagcgatattgatttgttggttactgtggccgtaaagcttgatgaaacgacgcggcgagcattgctcaatgatcttatggaggcttcggctttccctggcgagagcgagacgctccgcgctatagaagtcacccttgtcgtgcatgacgacatcatcccgtggcgttatccggctaagcgcgagctgcaatttggagaatggcagcgcaatgacattcttgcgggtatcttcgagccagccatgatcgacattgatctagctatcctgcttacaaaagcaagagaacatagcgttgccttggtaggtccggcagcggaggaattctttgacccggttcctgaacaggatctattcgaggcgctgagggaaaccttgaagctatggaactcgcagcccgactgggccggcgatgagcgaaatgtagtgcttacgttgtcccgcatttggtacagcgcaataaccggcaaaatcgcgccgaaggatgtcgctgccgactgggcaataaaacgcctacctgcccagtatcagcccgtcttacttgaagctaagcaagcttatctgggacaaaaagaagatcacttggcctcacgcgcagatcacttggaagaatttattcgctttgtgaaaggcgagatcatcaagtcagttggtaaatgaAAGCTT

aadA2

JM-62

ATTAATctacctctcactagtgaggggcggcagcgcatcaagcggtgagcgcactccggcaccgccaactttcagcacatgcgtgtaaatcatcgtcgtagagacgtcggaatggccgagcagatcctgcacggttcgaatgtcgtaaccgctgcggagcaaggccgtcgcgaacgagtggcggagggtgtgcggtgtggcgggcttcgtgatgcctgcttgttctacggcacgtttgaaggcgcgctgaaaggtctggtcatacatgtgatggcgacgcacgacaccgctccgtggatcggtcgaatgcgtgtgctgcgcaaaaacccagaaccacggccaggaatgcccggcgcgcggatacttccgctcaagggcgtcgggaagcgcaacgccgctgcggccctcggcctggtccttcagccaccatgcccgtgcacgcgacagctgctcgcgcaggctgggtgccaagctctcgggtaacatcaaggcccgatccttggagcccttgccctcccgcacgatgatcgtgccgtgatcgaaatccagatccttgacccgcagttgcaaaccctcactgatccgcatgcccgttccatacagaagctgggcgaacaaacgatgctcgccttccagaaaaccgaggatgcgaaccacttcatccggggtcagcaccaccggcaagcgccgcgacggccgaggtcttccgatctcctgaagccagggcagatccgtgcacagcaccttgccgtagaagaacagcaaggccgccaatgcctgacgatgcgtggagaccgaaaccttgcgctcgttcgccagccaggacagaaatgcctcgacttcgctgctgcccaaggttgccgggtgacgcacaccgtggaaacggatgaaggcacgaacccagtggacataagcctgttcggttcgtaagctgtaatgcaagtagcgtatgcgctcacgcaactggtccagaac

intI1

-35 -10

Pc promoter

cttgaccgaacgcagcggtggtaacggcgcagtggcggttttcatggcttgttatgactgtttttttgtacagtctatgcctcgggcatccaagcagcaagcgcgttacgccgtgggtcgatgtttgatgttatggagcagcaacgatgttacgcagcagggcagtcgccctaaaacaaagttagacatcatgagggaagcggtgaccatcgaaatttcgaaccaactatcagaggtgctaagcgtcattgagcgccatctggaatcaacgttgctggccgtgcatttgtacggctccgcagtggatggcggcctgaagccatacagcgatattgatttgttggttactgtggccgtaaagcttgatgaaacgacgcggcgagcattgctcaatgatcttatggaggcttcggctttccctggcgagagcgagacgctccgcgctatagaagtcacccttgtcgtgcatgacgacatcatcccgtggcgttatccggctaagcgcgagctgcaatttggagaatggcagcgcaatgacattcttgcgggtatcttcgagccagccatgatcgacattgatctagctatcctgcttacaaaagcaagagaacatagcgttgccttggtaggtccggcagcggaggaattctttgacccggttcctgaacaggatctattcgaggcgctgagggaaaccttgaagctatggaactcgcagcccgactgggccggcgatgagcgaaatgtagtgcttacgttgtcccgcatttggtacagcgcaataaccggcaaaatcgcgccgaaggatgtcgctgccgactgggcaataaaacgcctacctgcccagtatcagcccgtcttacttgaagctaagcaagcttatctgggacaaaaagaagatcacttggcctcacgcgcagatcacttggaagaatttattcgctttgtgaaaggcgagatcatcaagtcagttggtaaatgaAAGCTT

aadA2

JM-86

ATTAATctacctctcactagtgaggggcggcagcgcatcaagcggtgagcgcactccggcaccgccaactttcagcacatgcgtgtaaatcatcgtcgtagagacgtcggaatggccgagcagatcctgcacggttcgaatgtcgtaaccgctgcggagcaaggccgtcgcgaacgagtggcggagggtgtgcggtgtggcgggcttcgtgatgcctgcttgttctacggcacgtttgaaggcgcgctgaaaggtctggtcatacatgtgatggcgacgcacgacaccgctccgtggatcggtcgaatgcgtgtgctgcgcaaaaacccagaaccacggccaggaatgcccggcgcgcggatacttccgctcaagggcgtcgggaagcgcaacgccgctgcggccctcggcctggtccttcagccaccatgcccgtgcacgcgacagctgctcgcgcaggctgggtgccaagctctcgggtaacatcaaggcccgatccttggagcccttgccctcccgcacgatgatcgtgccgtgatcgaaatccagatccttgacccgcagttgcaaaccctcactgatccgcatgcccgttccatacagaagctgggcgaacaaacgatgctcgccttccagaaaaccgaggatgcgaaccacttcatccggggtcagcaccaccggcaagcgccgcgacggccgaggtcttccgatctcctgaagccagggcagatccgtgcacagcaccttgccgtagaagaacagcaaggccgccaatgcctgacgatgcgtggagaccgaaaccttgcgctcgttcgccagccaggacagaaatgcctcgacttcgctgctgcccaaggttgccgggtgacgcacaccgtggaaacggatgaaggcacgaacccagttgacataagcctgttcggttcgtaaactgtaatgcaagtagcgtatgcgctcacgcaactggtccagaacc

intI1

-35 -10

Pc promoter

ttgaccgaacgcagcggtggtaacggcgcagtggcggttttcatggcttgttatgactgtttttttgtacagtctatgcctcgggcatccaagcagcaagcgcgttacgccgtgggtcgatgtttgatgttatggagcagcaacgatgttacgcagcagggcagtcgccctaaaacaaagttagacatcatgagggaagcggtgaccatcgaaatttcgaaccaactatcagaggtgctaagcgtcattgagcgccatctggaatcaacgttgctggccgtgcatttgtacggctccgcagtggatggcggcctgaagccatacagcgatattgatttgttggttactgtggccgtaaagcttgatgaaacgacgcggcgagcattgctcaatgatcttatggaggcttcggctttccctggcgagagcgagacgctccgcgctatagaagtcacccttgtcgtgcatgacgacatcatcccgtggcgttatccggctaagcgcgagctgcaatttggagaatggcagcgcaatgacattcttgcgggtatcttcgagccagccatgatcgacattgatctagctatcctgcttacaaaagcaagagaacatagcgttgccttggtaggtccggcagcggaggaattctttgacccggttcctgaacaggatctattcgaggcgctgagggaaaccttgaagctatggaactcgcagcccgactgggccggcgatgagcgaaatgtagtgcttacgttgtcccgcatttggtacagcgcaataaccggcaaaatcgcgccgaaggatgtcgctgccgactgggcaataaaacgcctacctgcccagtatcagcccgtcttacttgaagctaagcaagcttatctgggacaaaaagaagatcacttggcctcacgcgcagatcacttggaagaatttattcgctttgtgaaaggcgagatcatcaagtcagttggtaaatgaAAGCTT

aadA2
